# Predictive biomarkers in precision medicine and drug development against lung cancer

**DOI:** 10.1186/s40880-015-0028-4

**Published:** 2015-07-02

**Authors:** Bingliang Fang, Reza J Mehran, John V Heymach, Stephen G Swisher

**Affiliations:** Department of Thoracic and Cardiovascular Surgery, The University of Texas MD Anderson Cancer Center, Houston, TX 77030 USA; Department of Thoracic and Head/Neck Medical Oncology, The University of Texas MD Anderson Cancer Center, Houston, TX 77030 USA

**Keywords:** Precision medicine, Personalized therapy, Epidermal growth factor receptor (EGFR), Anaplastic lymphoma kinase (ALK), Immunotherapy, Biomarkers, Drug development

## Abstract

The molecular characterization of various cancers has shown that cancers with the same origins, histopathologic diagnoses, and clinical stages can be highly heterogeneous in their genetic and epigenetic alterations that cause tumorigenesis. A number of cancer driver genes with functional abnormalities that trigger malignant transformation and that are required for the survival of cancer cells have been identified. Therapeutic agents targeting some of these cancer drivers have been successfully developed, resulting in substantial improvements in clinical symptom amelioration and outcomes in a subset of cancer patients. However, because such therapeutic drugs often benefit only a limited number of patients, the successes of clinical development and applications rely on the ability to identify those patients who are sensitive to the targeted therapies. Thus, biomarkers that can predict treatment responses are critical for the success of precision therapy for cancer patients and of anticancer drug development. This review discusses the molecular heterogeneity of lung cancer pathogenesis; predictive biomarkers for precision medicine in lung cancer therapy with drugs targeting epidermal growth factor receptor (*EGFR*), anaplastic lymphoma kinase (*ALK*), c-ros oncogene 1 receptor tyrosine kinase (*ROS1*), and immune checkpoints; biomarkers associated with resistance to these therapeutics; and approaches to identify predictive biomarkers in anticancer drug development. The identification of predictive biomarkers during anticancer drug development is expected to greatly facilitate such development because it will increase the chance of success or reduce the attrition rate. Additionally, such identification will accelerate the drug approval process by providing effective patient stratification strategies in clinical trials to reduce the sample size required to demonstrate clinical benefits.

## Background

A recent study analyzing the global disease burden revealed that lung cancer is the second leading cause of death in the United States and in most other high-income countries [1]. In China, lung cancer is the fifth leading cause of death, following stroke, ischemic heart disease, road injuries, and chronic obstructive pulmonary disease [1, 2]. Globally, the annual incidence of lung cancer is approximately 1.8 million, with an annual mortality of approximately 1.6 million [3, 4]. The 5-year overall survival (OS) rate for lung cancer patients in the United States has improved only moderately over the past four decades from 12.2% in 1975 to 17.8% in 2010, despite the use of many therapeutic modalities [5–7]. The 5-year OS rate of lung cancer patients in China is approximately 16.1% [8], which is close to that observed in the United States. Histologically, lung cancer is classified as small cell lung cancer (SCLC, approximately 15%) and non-small cell lung cancer (NSCLC, approximately 85%); the latter includes adenocarcinoma, squamous cell carcinoma, and large cell carcinoma. Tobacco smoking is the major cause for all types of lung cancer, particularly SCLC and squamous cell carcinoma [9]. Air pollution is another major cause of lung cancer, as a substantial number of lung cancer deaths in China and other East Asian countries may be attributed to fine particles in the air [10–12].

Small cell lung cancer is highly aggressive, and most (95%) SCLC patients are current or former smokers [13, 14]. Although the initial response rate to platinum-based chemotherapy and radiotherapy is high, recurrence and resistance occur inevitably in most SCLC patients, leading to 5-year OS rates of approximately 24% for patients with limited-stage SCLC and of less than 3% for those with metastases [5]. Fortunately, the incidence of SCLC in the United States has been declining over the past decades because of a decreasing prevalence of tobacco smoking [15]. For NSCLC, surgical resection is the standard care for patients with stage I disease, whereas surgical resection plus adjuvant therapy is used for patients with stages II–III disease. NSCLC patients at the advanced stages (stages IIIB–IV) are usually treated with chemotherapy, pathway-targeted therapies, and/or supportive medicine. Early diagnosis is the major factor proven to improve outcomes [16]; the 5-year OS rate is approximately 70% for patients with stage I NSCLC and drops to approximately 5% for patients with stage IV lung cancer [17]. Unfortunately, most lung cancers are diagnosed at an advanced stage and thus have a poor prognosis [9]. Early detection by low-dose computed tomography (CT) screening significantly reduces lung cancer mortality; nevertheless, approximately 96% of the positive results detected by such screening are false positives [18]. Moreover, the current estimated cost of screening to avoid one premature lung cancer death is approximately $240,000, thereby adding substantial expenditures to the health care system [19].

Advances in molecular profiling and targeted therapy have shown that subgroups of lung cancer patients are highly sensitive to some small-molecule inhibitors targeting key molecular nodes that drive carcinogenesis in those patients, such as epidermal growth factor receptor (EGFR) and anaplastic lymphoma kinase (ALK). These breakthrough discoveries have not only led to a new paradigm of biomarker-directed precision or personalized therapy but also greatly accelerated the development of novel anticancer drugs. It is now clear that mutations in cancer driver genes and alterations in gene expression and/or posttranscriptional modifications can all drastically affect treatment responses or clinical outcomes [20–22]. Moreover, the success of targeted anticancer therapy largely depends on biomarkers that can identify patient subgroups that may respond to specific therapeutic agents because alterations in a particular cancer driver usually exist in only a small subset of patients. This review discusses recent advances in predictive biomarker-directed precision medicine and drug development for lung cancers.

## Molecular heterogeneity in lung cancer

Genome-wide gene sequencing analyses have found that each lung cancer may have an average of approximately 150 somatic mutations that are expected to alter their protein products; this number is much higher than the average of 30–60 mutations observed in other solid tumors [23]. The high somatic mutation rates observed in lung cancer reflect the mutagenic roles of cigarette smoking in the pathogenesis of lung cancer. Indeed, the total number of point mutations in coding regions identified in lung cancer is approximately 10 times higher in smokers than in non-smokers [24]. Smoking also causes distinct changes in gene mutation and gene expression signatures in cancer tissues and normal lung tissues [25, 26]. Smoking-induced gene expression alterations observed in normal lung tissues can be transient because the majority of changes at the expression level can revert to non-smoker levels following smoking cessation [26]. Nevertheless, some of these expression changes are irreversible and permanent [26].

**Figure 1:**
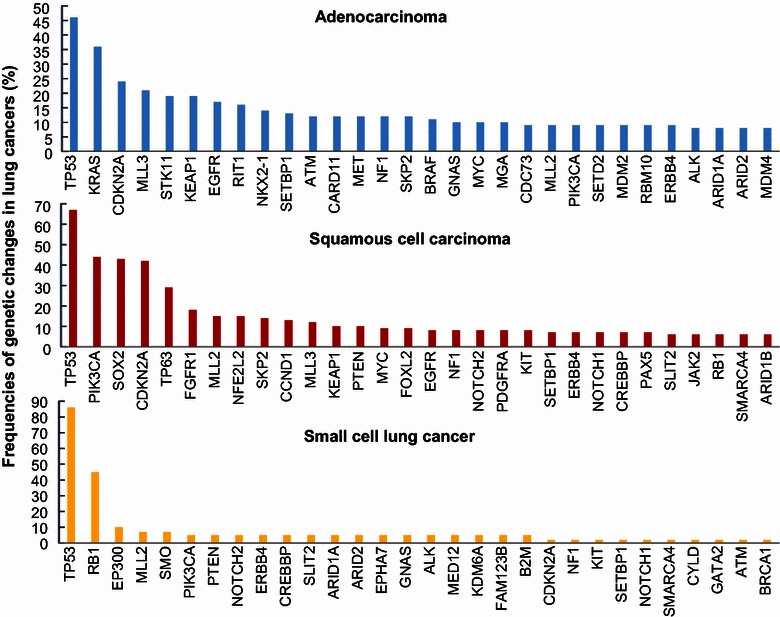
Frequencies of genetic alternations (mutations and copy number changes) in cancer driver genes. The frequencies for the top 30 mutated genes in lung adenocarcinoma, squamous cell carcinoma, and small cell lung cancer were retrieved from http://www.cbioportal.org. *TP53* tumor protein p53; *KRAS* Kirsten rat sarcoma viral oncogene homolog; *CDKN2A* cyclin-dependent kinase inhibitor 2A; *MLL3* mixed-lineage leukemia 3; *STK11* serine/threonine kinase 11; *KEAP1* kelch-like ECH-associated protein 1; *EGFR* epidermal growth factor receptor; *RIT1* Ras-like without CAAX 1; *NKX2*-*1* NK2 homeobox 1; *SETBP1* SET-binding protein 1; *ATM* ataxia telangiectasia-mutated; *CARD11* caspase recruitment domain family, member 11; *MET* MET proto-oncogene; *NF1* neurofibromin1; *SKP2* S-phase kinase-associated protein 2; *BRAF* v-Raf murine sarcoma viral oncogene homolog B1; *GNAS* GNAS complex locus; *MYC* v-myc avian myelocytomatosis viral oncogene homolog; *MGA* MAX dimerization protein; *CDC73* cell division cycle 73; *PIK3CA* phosphatidylinositol-4,5-biphosphate 3-kinase, catalytic subunit alpha; *SETD2* SET domain-containing 2; *MDM2* mouse double minute 2 homolog; *RBM10* RNA-binding motif protein 10; *ERBB4* erb-b2 receptor tyrosine kinase 4; *ALK* anaplastic lymphoma kinase; *ARID1A* AT rich interactive domain 1A; *SOX2* sex-determining region Y-related gene family 2; *FGFR1* fibroblast growth factor receptor 1; *NFE2L2* nuclear factor, erythroid 2-like 2; *CCND1* cyclin D1; *PTEN* phosphatase and tensin homolog; *FOXL2* forkhead box L2; *NOTCH2* notch 2; *PDGFRA* platelet-derived growth factor receptor, alpha polypeptide; *KIT* v-kit Hardy-Zuckerman 4 feline sarcoma viral oncogene homolog; *CREBBP* CREB-binding protein; *PAX5* paired box 5; *SLIT2* slit homolog 2; *JAK2* Janus kinase 2; *RB1* retinoblastoma 1; *SMARCA4* SWI/SNF-related, matrix-associated, actin-dependent regulator of chromatin, subfamily a, member 4; *EP300* E1A-binding protein p300; *SMO* smoothened; *EPHA7* EPH receptor A7; *MED12* mediator complex subunit 12; *KDM6A* lysine (K)-specific demethylase 6A; *FAM123B* family with sequence similarity 123B; *B2M* beta-2-microglobulin; *CYLD* cyclin dromatosis (turban tumor syndrome); *GATA2* GATA-binding protein 2; *BRCA1* breast cancer early onset.

**Figure 2:**
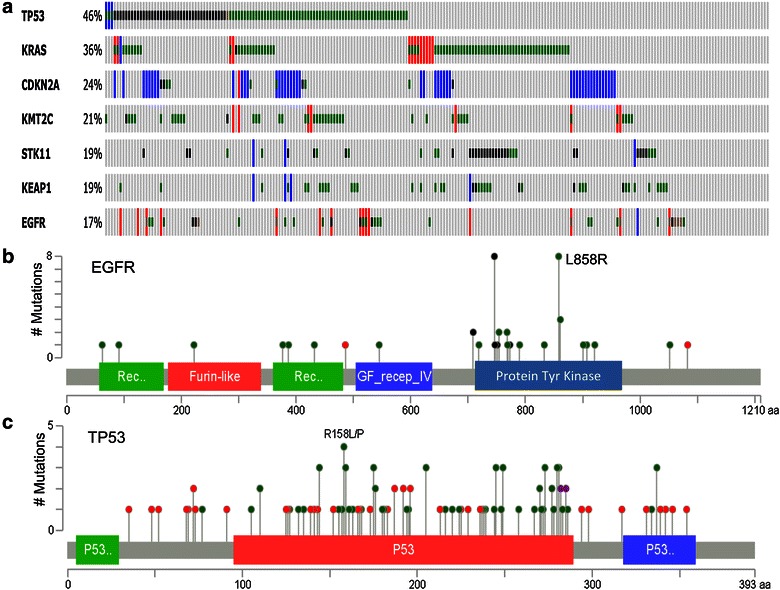
Molecular heterogeneity in lung adenocarcinoma. **a** The status of genetic alterations in 230 lung adenocarcinomas retrieved from The Cancer Genome Atlas (TCGA) database [28]. The mutation frequencies (%) are shown on the *left* of graph. Each *vertical line* represents a tumor. The *graph* shows mutations in the top seven cancer driver genes in lung adenocarcinoma. *Red* amplification, *blue* homozygous deletion, *green* missense mutation, *black* truncating mutation, *brown* in-frame mutation. **b**, **c** Mutations in *EGFR* and *TP53* in the same 230 adenocarcinomas. The *gray bars* represent the entire lengths of the EGFR and TP53 proteins, with the number of amino acids indicated at the *bottom* of each *gray bar*. The *green*, *red*, and *blue* *boxes* indicate protein domains. The *lines* and *dots* indicate the locations and frequencies of mutations detected in the *EGFR* and *TP53* genes. *Green* missense mutations, *red* nonsense or frameshift mutations, *black* in-frame deletions. The data were retrieved from http://www.cbioportal.org.

The results from genome-wide sequencing analyses for primary lung adenocarcinoma [24, 25, 27, 28], squamous cell cancer [29], SCLC [30, 31], and carcinoids or neuroendocrine tumors [32] have been reported recently by several groups. Information regarding genetic alterations (mutations and copy number changes), mRNA expression data, and protein/protein phosphorylation levels in lung adenocarcinoma, squamous cell cancer, and SCLC can be retrieved from The Cancer Genome Atlas (TCGA) databases and publically available datasets at the website http://www.cbioportal.org. Figure 1 shows the frequencies of cancer driver genes that are frequently mutated (including copy number changes) in lung adenocarcinoma, squamous cell cancer, and SCLC. Based on data retrieved from cBioPortal, tumor protein p53 (*TP53*) is the most frequently mutated cancer driver gene for all three histological types of lung cancer, with frequencies varying from 46% in adenocarcinoma to 86% in SCLC. Additionally, Kirsten rat sarcoma viral oncogene homolog (*KRAS*), cyclin-dependent kinase inhibitor 2A (*CDKN2A*), mixed-lineage leukemia 3 (*MLL3*), serine/threonine kinase 11 (*STK11*), kelch-like ECH-associated protein 1 (*KEAP1*), and *EGFR* are the top frequently mutated genes detected in lung adenocarcinomas. Phosphatidylinositol-4,5-biphosphate 3-kinase, catalytic subunit alpha (*PIK3CA*), sex-determining region Y-related gene family 2 (*SOX2*), *CDKN2A*, *TP63*, fibroblast growth factor receptor 1(*FGFR1*), and *MLL2* are the top mutant genes in squamous cell cancer; retinoblastoma 1 (*RB1*), E1A-binding protein p300 (*EP300*), *MLL2*, smoothened (*SMO*), and *PIK3CA* are the top mutant genes in SCLC. Our recent sequencing analysis of the exomes of 200 cancer-related genes found that mutations in *TP53*, *KRAS*, *MLL3*, SET domain-containing 2 (*SETD2*), AT rich interactive domain 1A (*ARID1A*), *PIK3CA*, and *ALK* were frequently detected in primary tumors and corresponding patient-derived xenografts of NSCLC [33]. Most of the mutations (93%) detected in the primary tumors were also detected in their corresponding patient-derived xenografts. Nevertheless, the numbers of mutations detected in each primary tumor varied greatly, and most tumors had mutations in more than two genes. The results from these molecular profiling studies clearly demonstrated heterogeneity in the molecular pathogenesis of lung cancer. As shown in Figure 2a, the mutational status of the top seven frequently mutated cancer driver genes varied greatly in 230 lung adenocarcinomas [28]. Moreover, mutations in those genes, particularly for tumor suppressor genes such as *TP53* (Figure 2b), are often widely distributed in the entire coding region. Evidence has shown that mutations in *TP53* can lead to either loss or gain of functions [34–38], and both may promote tumorigenesis through different mechanisms. Similarly, different mutations in *KRAS*, even if in the same codon such as G12C, G12V, and G12D mutations in the *KRAS* gene, may lead to different conformational changes in *KRAS* and have different effects on clinical outcomes and molecular pathway activation [39]. Ultimately, this molecular heterogeneity may affect treatment responses to therapeutics targeting different pathways.

Notably, however, current TCGA data are derived mostly from patients in Western countries. The mutational status in other ethnic populations may differ [40–42]. For example, activated *EGFR* mutations are detected in approximately 10–17% of lung adenocarcinoma patients in the United States and Europe [27, 43–46] but in approximately 30–65% of lung adenocarcinoma patients in Asia [47–50]. In contrast, *KRAS* mutations were detected in 35–50% of lung adenocarcinomas in Caucasian patients [28, 51] but in less than 5% of lung adenocarcinomas in Chinese patients [48, 49, 52]. Nevertheless, changes in molecular signaling can occur in the absence of gene mutations. Indeed, the activation of RAS signaling pathways can be detected in substantial numbers of primary tumors with wild-type *RAS* genes [53]. Increased expression of the *RAS* genes [54, 55], increased upstream signaling from tyrosine kinase growth factor receptors such as v-erbB2 avian erythroblastic leukemia viral oncogene homolog 2 (*HER2*) and *EGFR* [56, 57], and altered expression of microRNAs such as *let*-*7* [58, 59] may all contributes to the activation of RAS signaling pathways.

## Biomarker-directed precision medicine for lung cancer

### Anti-EGFR therapy

**Figure 3:**
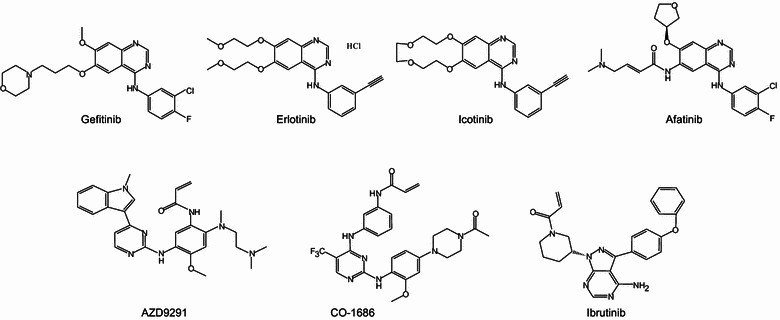
Chemical structures of anti-EGFR therapeutic agents.

Epidermal growth factor receptor, a major driver in lung tumorigenesis, has been extensively investigated as a target for anticancer therapy. As noted above, activating *EGFR* mutations are detected in substantial numbers of lung cancer patients and are more commonly observed in women and non-smokers [27, 43–46]. The finding that *EGFR* mutations in primary lung cancer are associated with sensitivity to EGFR inhibitors gefitinib and erlotinib [44, 60, 61] contributed greatly to the final approval of these therapeutics for the treatment of lung cancer. Subsequently, *EGFR* gene mutations have served as biomarkers in the clinic for the identification of responders to EGFR inhibitors. Both gefitinib and erlotinib (Figure 3) are the first choice for therapy in lung cancer patients whose tumors harbor *EGFR* mutations and are reported to significantly prolong progression-free survival (PFS) in patients with *EGFR*-mutant lung cancer [62–64]. Icotinib, which has a clinical efficacy similar to that of gefitinib but less adverse effects [65], is approved for the treatment of *EGFR*-mutant lung cancer in China.

As shown in Figure 2, mutations in *EGFR* are also heterogeneous. There are multiple locations in the *EGFR* gene where driver mutations can occur, and these mutations associate with differential sensitivity to EGFR inhibition. The most common *EGFR* mutations are in-frame deletions in exon 19 and L858R point mutations in exon 21. Together, these mutations account for approximately 85% of the *EGFR* mutations observed in lung cancer [46, 48, 66]; tumors with these mutations are highly sensitive to treatment with erlotinib, gefitinib, and the second-generation EGFR inhibitor afatinib [63, 67–69]. Tumors with other less common mutations, such as exon 18 mutations at G719 and exon 21 mutation L861Q, are also susceptible to treatment with EGFR inhibitors [60, 70]. Nevertheless, tumors with exon 20 in-frame insertions, which account for approximately 4–10% of all *EGFR* mutations, usually do not respond to treatment with EGFR inhibitors [71–73]. Although these insertional mutations activate *EGFR*, they do not have increased affinity for EGFR inhibitors, as is observed for other *EGFR* mutations [74].

However, despite initial dramatic responses to gefitinib and erlotinib in patients with *EGFR*-mutant lung cancers, acquired resistance inevitably occurs at a median of 10–13 months after treatment initiation [69, 75, 76]. A number of mechanisms for this acquired resistance have been identified, including a secondary T790M mutation at exon 20 of the *EGFR* gene [77–79], amplification of the MET proto-oncogene (*MET*) [80–82] or *HER2* gene [83, 84], mutations of the *KRAS* gene [85], activation of AXL receptor tyrosine kinase (AXL), V-Srcavian sarcoma (Schmidt-Ruppin A-2) viral oncogene homolog (SRC) kinases [86–88], and extracellular signal-regulated kinase (ERK) [89], loss of phosphatase and tensin homolog (*PTEN*) [90], and activation of the nuclear factor-κB (NF-κB) [91] or interleukin-6 receptor (IL-6R)/signal transducer and activator of transcription 3 (STAT3) pathway [92, 93]. Among these mechanisms, the most common cause of resistance observed clinically is acquisition of the *EGFR*T790M mutation, which is found in approximately 50% of patients [77, 80, 94]. A number of second-generation EGFR inhibitors, most of which are irreversible, inhibit two or more receptors in the EGFR family, and may have some activity in T790M-mutant cancers, have been evaluated in clinical trials [95–98]. Among those evaluated in clinical trials, only afatinib [96, 99] has been approved (in July 2013) by the United States Food and Drug Administration (FDA) for the treatment of metastatic NSCLC with *EGFR* mutations. Unfortunately, diarrhea and rash, the two most common adverse effects associated with a good response to the therapy, appear to be more severe in patients treated with afatinib than those treated with erlotinib or gefitinib [100]. More recently, the third-generation EGFR inhibitors AZD9291 [101] and CO-1686 [102, 103] have shown promising anticancer activity in T790M-mutant tumors. Our recent studies have revealed that ibrutinib, an irreversible inhibitor of Bruton tyrosine kinase (BTK) that was recently approved by the FDA for the treatment of mantle cell lymphoma and chronic lymphocytic leukemia [104, 105], can function as an EGFR inhibitor to selectively inhibit growth and induce apoptosis in *EGFR*-mutant NSCLC cells in vitro and in vivo, including erlotinib-resistant cells that harbor a T790M mutation [106]. Because ibrutinib was recently reported to be well tolerated when used in combination with other targeted therapeutics [107, 108], it may have advantages in combination therapy to overcome resistance caused by alternative or bypass survival signals from cooperative or parallel pathways, as is observed in T790M-negative resistant cells.

### Anti-ALK/ROS1 therapy

**Figure 4:**
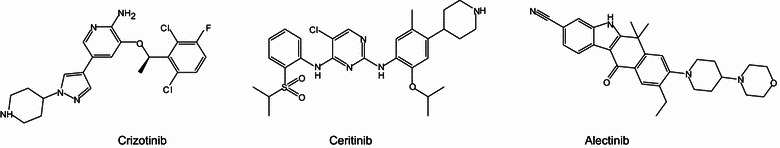
Chemical structures of anti-ALK/ROS1 therapeutic agents. *ROS1* c-ros oncogene 1 receptor tyrosine kinase.

The discovery of gene rearrangements between the echinoderm microtubule-associated protein-like 4 (*EML4*) gene and the *ALK* gene that lead to constitutive activation of ALK in NSCLC [109, 110] greatly contributed to the subsequent development and approval of crizotinib, a small-molecule inhibitor of ALK, c-ros oncogene 1 receptor tyrosine kinase (ROS1), and MET. *EML4*/*ALK* rearrangements are detected in approximately 4–5% of patients with NSCLC, mostly in young patients with adenocarcinoma who are non-smokers or light smokers [111–114]. *ALK* rearrangements were found to be mutually exclusive with *EGFR* and *KRAS* mutations, although they share common downstream signaling pathways with *EGFR* such as the RAS/RAF/MEK/ERK, PI3K/AKT/mTOR, and JAK/STAT pathways. Crizotinib (Figure 4) was originally developed as an MET inhibitor; however, crizotinib also inhibits ALK and ROS1 [115]. Soon after the first reports of *EML4*/*ALK* rearrangement and its role in lung cancer oncogenesis [109, 110], a diagnostic method for detecting *EML4*/*ALK* rearrangement by fluorescence in situ hybridization (FISH) was developed, and patients with *EML4*/*ALK* rearrangement-positive lung cancer were enrolled in clinical trials with crizotinib [114, 116]. Most of the patients with advanced ALK-positive NSCLC responded to crizotinib treatment, resulting in tumor shrinkage or disease stabilization. The objective response rate was approximately 60%, and the median PFS was 8–10 months [117, 118]. In comparison, conventional chemotherapy for the same type of patients achieved a response rate of approximately 20% and a PFS of 2–3 months [117, 118]. Subsequently, crizotinib was approved for the treatment of ALK-positive NSCLC in conjunction with an FDA-approved diagnostic assay, the Vysis ALK BreakApart FISH Probe Kit.

*ROS1* gene rearrangement and activation are detected in approximately 1–1.5% of NSCLC, predominantly in adenocarcinoma and in non-smokers, as is observed with *ALK* rearrangement [119–121]. However, *ROS1* and *ALK* rearrangements rarely occur in the same tumors. Because the kinase domains of ALK and ROS1 share 77% homology at the protein sequence level and crizotinib is highly active for both ALK and ROS1 [115], clinical investigation into the treatment of NSCLC patients with *ROS1* rearrangement has been pursued. The results indicated that crizotinib also has marked antitumor activity in patients with *ROS1*-rearranged advanced NSCLC, with an objective response rate of 72% and a median response duration of approximately 17.6 months [122]. The response duration in *ROS1* rearrangement-positive patients appears to be longer than that in ALK-positive patients (10–11 months). Similar to the diagnosis of *ALK* rearrangement, FISH and/or reverse transcription-polymerase chain reaction (RT-PCR) are used to identify patients with *ROS1* rearrangement [122]. Nevertheless, resistance to crizotinib eventually occurs in both ALK- and ROS1-positive patients.

Among the patients with ALK-positive NSCLC who acquired resistance to crizotinib, one-third had various secondary mutations in the ALK kinase domain and/or had *ALK* gene amplification [111]. Resistance can also be caused by aberrant activation of alternative or bypass pathways, such as marked v-kit Hardy-Zuckerman 4 feline sarcoma viral oncogene homolog (*KIT*) amplification and *EGFR* activation [123]. In some patients, multiple resistance mechanisms may exist simultaneously, indicating that combination therapy will be required to overcome resistance [124, 125]. Second-generation ALK inhibitors have been developed to overcome crizotinib resistance caused by mutations in the ALK kinase domain. Among these ALK inhibitors, ceritinib has been approved by the FDA for the treatment of ALK-positive NSCLC in the United States, and alectinib has been approved in Japan [126, 127] (Figure 4). Ceritinib is reported to be effective in patients with various resistant mutations. Of the patients who had received crizotinib previously, 56% responded to ceritinib treatment, including patients with central nervous system metastases [126, 128]. Nevertheless, ceritinib resistance has been observed in the clinic. Resistant cells obtained from patients treated with ceritinib revealed activation of the EGFR, mitogen-activated protein (MAP) kinase, and SRC pathways, and the cells were sensitive to combination therapies with inhibitors for EGFR, MAP kinase-ERK kinase (MEK), and SRC [129]. MET activation has been reported to cause resistance to alectinib because, as an ALK selective inhibitor, alectinib does not inhibit MET [130].

### Immunotherapy

The presence of tumor antigens derived from mutant proteins, dysregulated or overexpressed proteins, and viral oncogenic proteins has inspired intensive investigation into anticancer immunotherapy through various approaches [131]. The high mutation rates observed in lung cancers suggest that lung cancer cells may possess high levels of tumor antigens from mutant proteins and may therefore be vulnerable to immunotherapy. However, during malignant evolution, cancer cells obtain the ability to escape immune system recognition and destruction through various mechanisms, including inhibiting tumor antigen presentation by the down-regulation of class I major histocompatibility complex (MHC) molecule expression on the surface of tumor cells, recruiting immunosuppressive cells (such as regulatory T cells, myeloid-derived suppressor cells, and tumor-associated macrophages) to the tumor microenvironment, secreting immunosuppressive cytokines [such as IL-10 and tumor growth factor-β (TGF-β)], activating immune co-inhibitory checkpoint receptors [such as cytotoxic T lymphocyte-associated antigen-4 (CTLA-4) and programmed death-1 (PD-1)], and inhibiting immune co-stimulatory receptors (CD40, OX40, CD137, and CD28) [132–134]. Advances in the mechanistic characterization of tumor cell evasion of immunosurveillance have led to the development of various approaches that aim to mobilize the immune system for the elimination of malignant cells. Currently, the most advanced available therapeutics developed for lung cancer are monoclonal antibodies that target checkpoints of the immune regulation cascade.

Immune checkpoints regulate the amplitude and duration of immune responses, preventing damage to normal tissues from overactivation of the immune system [132–134]. The key players in the checkpoints include CTLA-4, lymphocyte activation gene-3 (LAG-3), and PD-1, as well as the PD-1 ligands PD-L1 and PD-L2. CTLA-4 is constitutively expressed in regulatory T cells but only transiently expressed in activated cytotoxic T cells. It shares common ligands, CD80 and CD86, with the co-stimulatory receptor CD28. The interaction of CD28 with these ligands leads to the full activation of cytotoxic T cells, whereas the interaction of CTLA-4 with the ligands prevents T cell activation. The up-regulation of CTLA-4 was detected in anergic lymphocytes, and the blockade of CTLA-4 with anti-CTLA-4 antibodies resulted in antitumor activity [135]. The anti-CTLA-4 antibody ipilimumab is approved for the treatment of melanoma [136] and is currently in clinical trials for the treatment of NSCLC. PD-1 (also known as PDCD1 or CD279) is expressed in T, B, and natural killer (NK) cells. The interaction of PD-1 with its ligands, PD-L1 (also called B7-H1 or CD274) and PD-L2 (B7-DC), suppresses T cell activation and promotes T cell apoptosis [137–139], leading to immune evasion. The expression of PD-L1 in lung cancer is detected in 25–50% of NSCLC cases, depending on the assays used to detect its protein or mRNA [140], and is associated with the presence of *EGFR* mutations in NSCLC [141]. EGFR activation induced PD-L1 expression in bronchial epithelial cells, whereas treatment with EGFR inhibitors reduced PD-L1 expression [142]. PD-1 monoclonal antibodies (nivolumab and pembrolizumab) have been approved for the treatment of melanoma [143–146]. The objective response rates in melanoma patients are approximately 26–40% [143, 144, 146]. Nivolumab was approved for the treatment of NSCLC in the United States in March 2015 [147] and was found to be highly active in treating Hodgkin’s lymphoma [148]. Monoclonal antibodies for PD-L1 are also under intensive clinical evaluation for the treatment of NSCLC and other solid tumors [149, 150]. The objective response rates in patients with advanced NSCLC are approximately 17–20% in patients receiving ≥1 mg/kg of anti-PD1 or anti-PD-L1 antibodies every 2–3 weeks, and the responses may last 1 year or longer [147, 149–151]. The response rates to both anti-PD1 and anti-PD-L1 antibodies were significantly higher in PD-L1-positive patients than in PD-L1-negative patients [150, 151]. In a trial with an anti-PD1 antibody, 9 (36%) of 25 PD-L1-positive patients responded to the treatment, whereas none of 17 PD-L1-negative patients had an objective response [151]. The study with the anti-PD-L1 antibody also found that responses were often observed in patients with tumors expressing high levels of PD-L1, especially if PD-L1 was expressed by tumor-infiltrating immune cells. The expression of CTLA-4, the absence of fractalkine (CX3CL1) [150], and the presence of pre-existing CD8^+^ T cells distinctly located at the invasive tumor margin [145] were also found to be associated with treatment responses to the PD1/PD-L1 blockage antibodies. These results demonstrated the feasibility of using the expression of PD-L1 and the presence of immune cells in tumor sites for patient stratification for PD1/PD-L1 blockade therapy. Nevertheless, larger clinical trials will be required to further validate the associations between PD-L1 expression and improved clinical outcomes.

## Predictive biomarkers in anticancer drug development

Anticancer drug development has a high failure or attrition rate, primarily because of a lack of efficacy, inability to identify responders, and intolerable toxic effects [152, 153]. Only approximately 5% of anticancer agents evaluated in human studies between 1991 and 2000 were successfully approved for clinical applications [152, 154]. The majority (80%) of phase III failures were caused by a lack of efficacy [152, 154–156] that resulted from a lack of efficacy proof-of-concept in humans and a lack of objective biomarkers capable of reporting such efficacy [152, 154]. The development of the anti-EGFR drugs gefitinib and erlotinib and the anti-ALK/ROS1 drugs crizotinib and ceritinib has provided excellent examples of using predictive biomarkers and patient stratifications in clinical trials to increase the chance of success or to reduce the attrition rate in anticancer drug development. Notably, both gefitinib [64, 157, 158] and erlotinib [159, 160] failed to show a benefit in randomized phase III trials with unselected patient populations. Similarly, the successes of the v-Raf murine sarcoma viral oncogene homolog B1 (BRAF) inhibitor vemurafenib [161, 162] and the poly(ADP-ribose) polymerase (PARP) inhibitor olaparib [163–165] in anticancer therapy relied on biomarker-based patient stratifications in clinical trials. In fact, the inability to identify cancers that are most likely to respond to a treatment is a major challenge in anticancer drug development, not only leading to failure to demonstrate the potential benefit of a promising anticancer agent [64, 166] but also exposing patients to the risks of ineffective treatments. Indeed, a lack of predictive biomarkers for patient stratification has halted further development of many other investigational drugs [167–170]. Thus, reliable predictive biomarkers are crucial to the success of anticancer drug development, and the simultaneous development of predictive biomarkers will be highly desirable for promising anticancer drug candidates.

The identification of predictive biomarkers can be relatively straightforward if the therapeutic targets are themselves the cancer drivers and if the cancer cells are addicted to the presence of those targets for survival. As in the cases of EGFR, ALK, and ROS1, the constitutive activation of such targets is the driving cause of a subset of NSCLC and is required for the survival of those cancer cells; therefore, the presence of constitutive activating mutations or gene rearrangement can be explored as predictive biomarkers for patient stratification. Nevertheless, cancer cells can be killed indirectly through synthetic lethality between the cancer drivers and their synthetic lethal partners, as has been demonstrated using the PARP inhibitor olaparib to eliminate breast cancer early onset (*BRCA*)-mutant ovarian and breast cancer cells [165, 171, 172]. In this case, loss-of-function mutations in the *BRCA1* and *BRCA2* genes or other functional deficiencies in the DNA repair pathways will serve as predictive biomarkers for response to olaparib and other PARP inhibitors [173]. Synthetic lethality is a lethal phenotype caused by the combined effects of mutations in two or more genes. This concept has recently been exploited for the development of novel genotype-selective anticancer agents and/or the identification of novel therapeutic targets [173–176]. This approach could be useful for the indirect targeting of cancer drivers such as genetic changes in *TP53*, *STK11*, *RB1*, and *RAS* genes that are nondruggable or difficult to target using small molecules. Notably, a study conducting short hairpin RNA (shRNA) library screening for genes that may have synthetic lethal interactions in the oncogenic *KRAS* gene in the colon cancer cell line DLD-1 and its isogenic derivatives led to the identification of approximately 370–1,600 *KRAS* synthetic lethal genes, depending on the stringencies of statistical analyses [177], demonstrating the diversity of biological processes or pathways regulated by *KRAS*. The molecular pathology of RAS-mediated oncogenesis is further complicated by the fact that cancer cells with different mutations in *KRAS*, even if in the same codon (such as G12C, G12V, and G12D mutations), may lead to different conformational changes in KRAS, have different effects on clinical outcomes, and activate different molecular and metabolic pathways [39, 178]. Moreover, a substantial number of *KRAS*-mutant tumors have mutations in other cancer driver genes such as *TP53*, *CDKN2A*, and *STK11*. Studies in transgenic mice have found that activating mutations in *KRAS* predispose mice to early-onset lung adenocarcinoma [179, 180]. Nevertheless, such tumors do not have an invasive or metastatic phenotype [181]. Metastasis will occur only when additional genetic changes, such as *Tp53* mutations or *Stk11* deletion, are introduced [182, 183]. Finally, the RAS activation signature is observed in approximately 25% of *KRAS* wild-type cancers [53] because increased RAS activity in human cancers can result from *RAS* gene amplification [184, 185] or overexpression [55], an increase in upstream signals from tyrosine kinase growth factor receptors such as HER2 and EGFR [56, 57], or a decrease in the expression of microRNAs such as let-7 family members [58].

The above-discussed situations impose considerable challenges on the identification of predictive biomarkers for anti-RAS therapeutics developed through synthetic lethality screening. Substantial efforts will be required to identify predictive biomarkers for such therapeutic agents. Through synthetic lethality screening using a chemical library for isogenic cell lines with and without oncogenic *KRAS*, we previously reported a small molecule, designated oncrasin-1, that induces apoptosis in several *KRAS*-mutant lung cancer cell lines [186]. The in vitro and in vivo activities were optimized by the synthesis and evaluation of analogues [187–190]. The mechanistic characterization revealed that these compounds induce oxidative stress, activate c-Jun N-terminal kinase (JNK), and inhibit RNA polymerase II, STAT3, and cyclin D1 [187, 188, 191–194]. However, a predictive biomarker for oncrasin compounds remained elusive until our recent discovery that such compounds require the expression of a sulfotransferase (SULT), SULT1A1, in cancer cells for their anticancer activity and that the expression of SULT1A1 is capable of predicting the responses to oncrasin compounds [195]. SULT1A1 is a biotransformation enzyme that bioactivates several pro-carcinogens [196–202] and some anticancer drugs such as tamoxifen [203]. The identification of SULT1A1 as a predictive biomarker for oncrasin compounds shed light on mechanisms of the selectivity and, possibly, the toxicity of these compounds [204]. The process of identifying this predictive biomarker underscores the importance of activity characterization in a large set of molecularly annotated cancer cell lines and of collaborations between biostatisticians and molecular biologists. Statistical analysis of associations between the activity of NSC-743380 (oncrasin-72) and the gene expression profile in the NCI-60 cell line panel led to the identification of a number of candidate genes, and subsequent molecular characterizations demonstrated the causal relationship between the expression of SULT1A1 and the anticancer activity of NSC-743380, validating the feasibility of using SULT1A1 as a predictive marker for those compounds [195]. Larger panels of molecularly annotated cancer cell lines have also been used by other groups for the identification of predictive biomarkers for larger sets of anticancer drugs [150, 205–209]. Ultimately, rigorous validation of causal relationships between the sensitivity and the biomarker will be essential for successful clinical translations.

## Conclusion

The diversity found in the molecular pathogenesis of lung cancer demonstrates that conventional histology-based classifications of the disease are not sufficient to provide guidance for treatment decisions in the clinic. Classifications based on molecular pathology are required for precision therapy. The successful development of anti-EGFR and anti-ALK/ROS1 therapeutics has proven the critical role of predictive biomarkers in anticancer drug development because the ability to identify patient subpopulations who may benefit from a drug candidate will directly affect the design and patient selection of clinical trials, dramatically reduce the sample size required to reveal potential benefits, and increase the chance of success. It is expected that more efforts will be devoted to the identification of predictive biomarkers and the development of effective assays for biomarkers during drug development. These efforts will likely be rewarded by increased success rates when the drug candidates enter clinical trials. Advances in technologies, such as next-generation sequencing and digital polymerase chain reaction, will also facilitate the detection of changes in genotypes and/or in DNA or RNA levels and the development of effective assays for biomarkers. On the other hand, biomarker-directed patient stratification has changed the clinical practice of anticancer therapy and has substantially improved treatment efficacy by providing guidance for the selection of optimal treatment for some lung cancer patients. However, biomarker-directed personalized therapy can currently benefit only a limited number of patients because most of the currently available predictive biomarkers occur infrequently [24, 27, 210] and because response-predictive biomarkers are not available for most first-line anticancer therapeutics. Thus, alternative approaches will be required to identify the appropriate therapeutics for a particular patient. Such patient-oriented approaches may require direct testing of the drug sensitivity of a patient’s cancer cells to various available anticancer therapeutics rather than predicting drug sensitivity based on the presence or absence of predictive biomarkers. Some recent studies have shown the feasibility of testing for the treatment responses of patient-derived tumor xenografts [211, 212] and/or primary cell cultures [213] to identify effective treatment regimens. Nevertheless, technology improvements and rigorous validations are required before such techniques can be used commonly in clinical practice.
